# Predictors and outcomes of recurrent bloodstream infection in hematological malignancies: a multicenter real-world study

**DOI:** 10.3389/fcimb.2026.1874798

**Published:** 2026-09-01

**Authors:** Yishu Tang, Yuhan Yan, Kai Zhao, Qian Cheng, Liwen Wang, Yulian Xiao, Zheng Fang, Yajing Wei, Xin Li

**Affiliations:** 1 Department of Hematology, The Third Xiangya Hospital, Central South University, Changsha, Hunan, China; 2 Department of Emergency Department, The Third Xiangya Hospital, Central South University, Changsha, Hunan, China

**Keywords:** Charlson comorbidity index, febrile episodes, hematological malignancies, mortality, recurrent bloodstream infections

## Abstract

Recurrent bloodstream infection (RBSI) is a life-threatening complication in patients with hematological malignancies (HMs), yet the determinants of recurrence remain poorly defined. We aimed to characterize the incidence, outcomes, and time-specific predictors of RBSI in HMs, and further explored the prognostic factors of RBSI. We performed a real-world study involving HM patients with BSI from three academic teaching hospitals during January 2010 to August 2023. Clinical characteristics associated with RBSI were assessed at the initial BSI and during subsequent febrile episodes after apparent resolution of the initial infection. Among 1,153 patients with HMs and BSI, 173 (15.0%) developed RBSI, whose mortality increased progressively with recurrent episodes, from 10.7% to 19.0% after the first recurrence, and to 30.5% after the second recurrence (P<0.01). At the initial BSI, a Charlson Comorbidity Index (CCI) >3 was associated with subsequent recurrence. During later febrile episodes, CCI >3, prior antibiotic exposure, acute respiratory failure, and platelet count <10×10^9^/L were enriched in patients with RBSI (all P<0.05). These findings support CCI >3 at initial BSI and adverse clinical features during subsequent fever may facilitate early identification of patients at high risk of recurrence and support more timely, targeted management.

## 1 Introduction

Bloodstream infection (BSI) is a severe systemic infection that may rapidly progress to sepsis, septic shock, or multiple organ dysfunction syndrome and remains associated with substantial mortality (Giannella et al., 2020; Kern and Rieg, 2020). RBSI, usually defined as a new episode of BSI occurring after clinical resolution of a previous episode, which occurs in approximately 8.0%–15.0% of patients with BSI and is associated with worse outcomes than the initial episode (McCarthy and Paterson, 2017; Froding et al., 2020; Bock et al., 2023; Abbara et al., 2024), poses an additional therapeutic and prognostic challenge (Gradel et al., 2017; Kim et al., 2021; Bock et al., 2023). Owing to chemotherapy-induced neutropenia, mucosal barrier injury, and repeated exposure to broad-spectrum antibiotics, which together facilitate opportunistic and resistant infections, patients with HMs are highly susceptible to RBSI (Reed et al., 2020; Chinese Society of Hematology, Chinese Medical Association; Chinese Hematology Association, Chinese Medical Doctor Association, 2020a; Al Beesh et al., 2024; Hu et al., 2024).

Limited single-center studies summarized the incidence of RBSI and the description of pathogen characteristics in patients with leukemia and recipients of allogeneic hematopoietic stem cell transplantation (Cattaneo et al., 2014; Akhmedov et al., 2023). Large multicenter studies evaluating the clinical features, outcomes, and stage-specific risk factors of RBSI in the broader HM population are lacking, particularly with respect to factors identifiable at the initial BSI episode or during subsequent febrile events that may enable early recognition of recurrence, and improve the prognosis of HM patients with RBSI.

Therefore, we conducted a multicenter real-world study in the hematology departments of three tertiary hospitals in Central-South China over a 14-year period. Patients with HMs and BSI were classified according to whether recurrent episodes occurred. We compared their clinical and microbiological characteristics and explored factors associated with recurrence at different stages of the clinical course, with the aim of improving risk stratification and informing individualized anti-infective management.

## 2 Patients and methods

### 2.1 Setting and study design

This multicenter real-world study was approved by the Institutional Review Board of the Third Xiangya Hospital, Central South University, China (Approval No. K26106). The requirement for informed consent was waived because only de-identified routinely collected clinical data were used.

**Figure 1:**
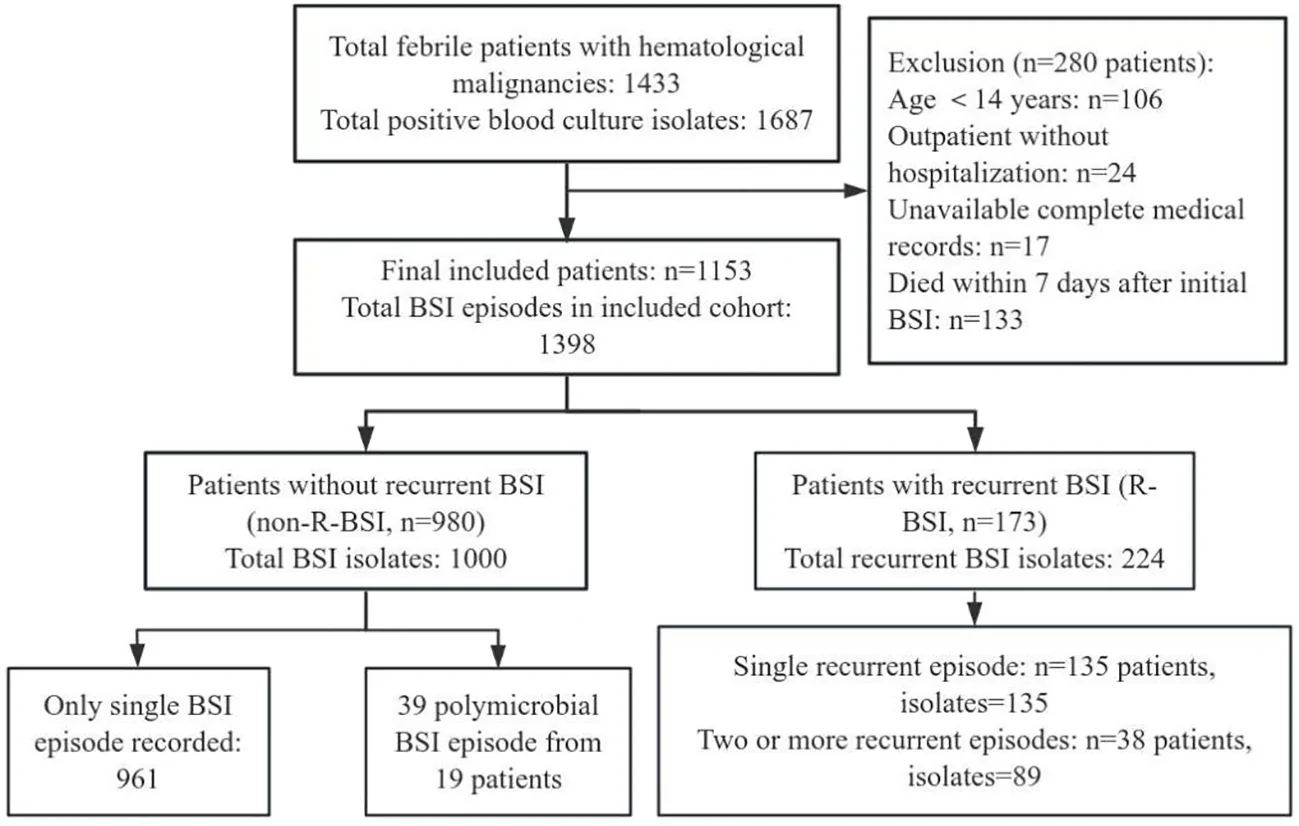
Flow chart of patient screening and cohort grouping.

Data were extracted from the electronic medical records of patients with hematological malignancies admitted to three academic teaching hospitals in Hunan Province, China, between January 2010 and August 2023. The case selection process is shown in **Figure 1**. Ultimately, 1,398 BSI episodes in 1,153 patients were included in the final analysis. Eligible patients were those aged ≥14 years with hematological malignancies and at least one confirmed BSI episode during hospitalization. Patients were excluded if they had outpatient presentation without admission, unavailable medical records, or died within 7 days after the initial BSI episode. The latter criterion was applied to ensure sufficient observation time for assessment of recurrence. Patients who died within 7 days of initial BSI were excluded to ensure adequate follow-up for detecting R-BSI, though this may introduce survival selection bias by excluding the sickest patients. A pre-specified sensitivity analysis was performed to address this bias, as detailed in Statistical Analysis. The primary outcome was all-cause in-hospital mortality within 30 days after BSI onset.

### 2.2 Definitions

Fever was defined as a single oral temperature ≥ 38.3 °C (axillary temperature ≥ 38.0 °C), or an oral temperature ≥ 38.0 °C (axillary temperature ≥ 37.7 °C) lasting for more than 1 hour (Cattaneo et al., 2014). The BSI was defined as an infection manifested by (1) the presence in at least two blood culture of bacteria other than skin contaminants (i.e., diphtheroids, Bacillus species., Propionibacterium acnes, CoNS, micrococci); (2) the presence of any bacterial species in at least one consecutive blood cultures in patients with the presence of an intravascular catheter and the initiation of targeted antimicrobial therapy were required for the diagnosis, as well as at least 1 of the following findings: temperature of >38.0 °C or <36.0 °C, chills, and/or systolic blood pressure of <90 mmHg, if not considered to be contaminated (Tang et al., 2018). Anti-infective treatment was administered according to contemporary clinical guidelines (Chinese Society of Hematology, Chinese Medical Association; Chinese Medical Doctor Association, Hematology Branch, 2020b). The date of the first positive blood culture was defined as the onset date of the initial BSI episode. Recurrent BSI (RBSI) was defined as a new episode of BSI, with either the same or a different pathogen, occurring more than 72 hours after clinical improvement of the preceding episode and discontinuation of antibiotic therapy (Akhmedov et al., 2023). Briefly, clinical improvement was confirmed by sustained fever resolution, relief of infection-associated symptoms, stable hemodynamic status, and amelioration of inflammatory biomarkers. Considering that prolonged fever and persistent neutropenia are common in this patient population, we did not require full recovery of neutrophil counts to define clinical improvement. Subsequent fever defined as a new febrile event occurring after clinical improvement of the initial BSI and following a minimum of 72 hours off antimicrobial therapy. Polymicrobial BSI was defined as isolation of two or more pathogens from blood cultures within 72 hours (Akhmedov et al., 2023). Hospital-acquired infection were defined as previously described (Tang et al., 2018).

The following variables were collected: demographic characteristics, underlying hematological malignancy, disease status, comorbidities, causative pathogens, multidrug-resistant organisms, antimicrobial exposure and therapy, and clinical severity. Disease status was assessed using the most recent bone marrow biopsy and categorized as remission, relapsed malignancy, or uncontrolled malignancy, as previously described (Tang et al., 2020). The Pitt bacteremia score was calculated at the time of fever and incorporated temperature, blood pressure, mental status, requirement for mechanical ventilation, and cardiac arrest (Tang et al., 2018). Neutropenia and profound neutropenia were defined as an absolute neutrophil count of <500 cells/mm³ and <100 cells/mm³, respectively (Chinese Society of Hematology, Chinese Medical Association; Chinese Medical Doctor Association, Hematology Branch, 2020b). Comorbidity burden was quantified using the Charlson Comorbidity Index (CCI). Diagnoses were identified via ICD-10 codes according to the Sundararajan algorithm, and the original weighting system described by Charlson et al. (1987) was used. Based on the characteristics of our cohort and receiver operating characteristic curve analysis, prolonged neutropenia was defined as neutropenia lasting >21 days. Acute respiratory failure and acute renal failure were defined as previously described (Tang et al., 2018). Multidrug resistance (MDR) was defined as acquired non-susceptibility to at least one agent in three or more antimicrobial categories (Magiorakos et al., 2012). Antibiotic exposure was defined as receipt of any systemic antimicrobial therapy for >48 hours within the preceding month (Tang et al., 2018). Inappropriate initial antimicrobial therapy within 72 hours (72h-IIAT) was defined as an antimicrobial regimen administered during the first 72 hours after suspected BSI that lacked *in vitro* activity against the pathogen subsequently identified by culture and susceptibility testing (Tang et al., 2020). Pathogen identification and antimicrobial susceptibility testing were performed using standard microbiological methods and an automated system (VITEK 2; bioMérieux, Durham, NC, USA), according to Clinical and Laboratory Standards Institute guidelines (Maschmeyer et al., 2015).

### 2.3 Statistical analysis

Statistical analyses were performed using SPSS Statistics version 26.0 (IBM Corp., Armonk, NY, USA) and R software version 4.5.1 (R Foundation for Statistical Computing, Vienna, Austria). Categorical variables are presented as counts and percentages, and continuous variables as mean ± SD or median (IQR), as appropriate. Patients were divided into recurrent and non-recurrent groups according to the occurrence of RBSI. To evaluate risk factors for recurrence and early warning indicators, analyses were conducted at two time points: initial BSI onset and subsequent fever after recovery from the initial episode. Comparisons between groups were performed using appropriate tests according to data type and distribution. All variables yielding P < 0.10 in univariate analyses and parameters with recognized clinical significance were incorporated into multivariable models. Two multivariable regression models were applied to address distinct research objectives. Cox proportional hazards regression was used to identify risk factors for the first episode of RBSI following initial BSI. The time interval from initial BSI to recurrent infection varied substantially across individuals, and hazard ratios were reported for this censored time-to-event outcome. Multivariable logistic regression was adopted for two analyses: it modelled binary infection status within each discrete febrile episode to predict confirmed RBSI (instead of evaluating time elapsed since initial BSI), and it also assessed predictors of in-hospital mortality. Odds ratios are presented for both logistic regression analyses. A pre-planned sensitivity analysis was conducted to assess bias from excluding patients dying within 7 days post initial BSI. These early-death patients were assigned to the non-R-BSI group, and all Cox models were re-fitted to test result robustness. Baseline features of early-death patients are provided in **Supplementary Tables**. To explore clinical predictors of RBSI at the onset of subsequent fever following initial BSI remission, we restricted the analysis to the subgroup of patients who experienced post-infection febrile episodes. This subgroup contained 92 patients who did not develop RBSI and 135 patients with confirmed RBSI. Given the clinical complexity of patients experiencing two or more recurrent BSI episodes, 38 eligible patients were excluded from this analysis. All baseline confounding factors were adjusted for in subsequent multivariable regression models to balance intergroup differences. All tests were two-sided, and *P* < 0.05 was considered statistically significant.

## 3 Results

### 3.1 RBSI association with adverse clinical outcomes

Of the initial 1,433 patients with hematological malignancies, 1,153 were included in the primary analysis. A total of 133 patients who died within 7 days following the initial BSI episode were excluded. These excluded patients exhibited substantially poorer baseline clinical profiles: they were more likely to be older than 55 years (34.6%), had a higher prevalence of relapsed or uncontrolled hematological malignancies (88.0%), and experienced higher rates of vasopressor support (63.2%) and acute respiratory failure (63.2%), as summarized in the **Supplementary Table 4**. Of the 1,153 patients with hematological malignancies, 173 (15.0%) developed recurrent bloodstream infection (RBSI), accounting for 224 recurrent isolates. Among these patients, 135(78.0%) experienced one recurrence, 31(17.9%) experienced two recurrences, and 7 (4.0%) experienced three or more recurrences (**Figure 1**). The median interval from initial BSI to recurrence was 85 days (range, 4–2,631 days).

**Figure 2:**
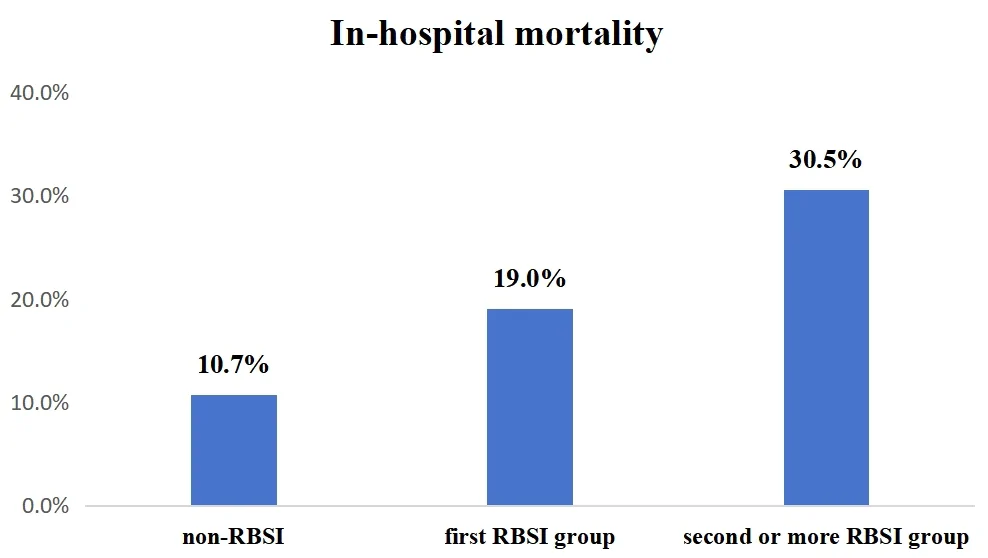
Crude in-hospital mortality stratified by the number of BSI episodes. RBSI: recurrent bloodstream infection.

RBSI was associated with significantly increased in hospital mortality compared with non-recurrent BSI (21.4% vs. 10.7%, *P* < 0.05). A clear exposure–response relationship was observed between recurrence frequency and mortality (*P* for trend < 0.001): mortality was 10.7% (105/980) in patients without recurrence, 19.0% (26/135) in those with one recurrent episode, and 30.5% (11/36) in those with at least two recurrent episodes (**Figure 2**). As shown in **Table 1**, patients with RBSI group more frequently included younger patients, those with CCI >3, and those with profound neutropenia. Prior antibiotic exposure and time to appropriate antimicrobial therapy did not differ significantly between the two groups.

**Table 1:** Clinical features associated with RBSI and non-RBSI groups at the initial BSI.

Variables	non-RBSI group (n, %) n=980	RBSI group (n, %) n=173	P
Demographic information			
Age (mean ± SD)	42.32 ± 15.07	37.88 ± 15.05	<0.001
Age >55 years	219 (22.3)	25 (14.5)	0.019
Male sex	534 (54.5)	98 (56.6)	0.599
Underlying malignancies			
Acute myeloid leukemia	496 (50.6)	96 (55.5)	0.237
Acute lymphoblastic leukemia	259 (26.4)	56 (32.4)	0.106
Lymphoma	99 (10.1)	13 (7.3)	0.289
Other malignancies	126 (12.8)	8 (4.6)	0.032
HSCT	37 (4.2)	2 (1.3)	0.083
Disease status			
Relapsed or uncontrolled	632 (64.5)	103 (59.5%)	0.212
Risk factors			
Charlson Comorbidity index >3	113 (11.5)	31 (17.9)	0.019
Pitt bacteremia score ≥4	172 (17.6)	28 (16.2)	0.662
MASCC<21	575 (65.8)	109 (70.8)	0.226
Neutropenia	859 (87.7)	160 (92.5)	0.067
Profound neutropenia	686 (77.0)	135 (85.4)	0.018
Prolonged neutropenia	302 (30.8)	54 (31.2)	0.917
Previous chemotherapeutics	833 (85.0)	160 (92.5)	0.009
Receipt of corticosteroids	398 (45.5)	80 (51.9)	0.141
Hospital infection	545 (78.9)	99 (84.6)	0.153
central venous catheters	384 (43.9)	68 (44.2)	0.960
urinary catheters	40 (4.6)	7 (4.5)	0.986
Complications of dysfunctional organ systems			
Use of vasopressors	138 (14.1)	23 (13.3)	0.783
Acute respiratory failure	109 (11.1)	23 (13.3)	0.408
Renal insufficiency	32 (3.7)	3 (1.4)	0.139
Antibiotic therapy			
Prior antimicrobial exposure	652 (66.5)	123 (71.1)	0.238
Use sensitive antibiotic	852 (86.9)	148 (86.0)	0.329
72h-IIAT	297 (30.3)	47 (27.2)	0.406
Time to appropriate antibiotic therapy, mean days	2.67	2.62	0.856
Bacteria resistance			
MDR-GNB	508 (59.0)	77 (52.7)	0.185
ESBL-GNB	166 (34.7)	32 (39.0)	0.202
CR-GNB	35 (4.7)	4 (3.9)	0.695
Laboratory parameters			
Hemoglobin <70g/dL	756 (77.1)	130 (75.1)	0.566
Platelet count <10×10^9^/L	516 (52.7)	103 (59.5)	0.094
Albumin <30g/L	497 (50.7)	80 (46.2)	0.278
AST >120U/L	76 (7.8)	13 (7.5)	0.913
TBil >34.2µmol/L	125 (12.8)	25 (14.5)	0.541
PT >14s	226 (23.1)	34 (19.7)	0.323

Footnote: P < 0.05 indicates statistical significance. MASCC, Multinational Association for Supportive Care in Cancer; 72h-IIAT, 72-hour inappropriate initial antimicrobial therapy; MDR-GNB, multidrug-resistant Gram-negative bacteria; ESBL-GNB, extended-spectrum β-lactamase-producing Gram-negative bacteria; CR-GNB, carbapenem-resistant Gram-negative bacteria; AST, aspartate aminotransferase; TBil, total bilirubin; PT, prothrombin time.

### 3.2 Risk factors of recurrent bloodstream infection at the initial episode

Since recurrent bloodstream infection leads to higher mortality, we explored the risk factors for its occurrence. Multivariable analysis showed that CCI >3 was the only independent predictor of RBSI in patients with hematological malignancies (*P* = 0.041; **Table 2**). Comparison of clinical features between initial and recurrent episodes showed that patients with RBSI had persistently unresolved or even worsening risk factors at recurrence (**Supplementary Tables 2**, **3**). Notably, vasopressor use increased significantly from the initial episode to the recurrent episode (14.8% vs. 23.7%, *P* = 0.034), suggesting aggravated organ dysfunction. In contrast, patients without RBSI showed marked improvement in clinical risk factors during subsequent infection episodes. To validate the robustness of our primary findings, we performed a sensitivity analysis in which patients who died within 7 days following their initial BSI were assigned to the non-R-BSI cohort. The Sensitivity analysis confirmed that a CCI > 3 measured at initial BSI remained independently associated with the subsequent development of R-BSI (**Supplementary Table 5**).

**Table 2:** Multivariable Cox regression analysis of factors associated with the time to RBSI after the initial BSI.

Factor	Hazard ratio	95% confidence interval	P value
Age >55 years	0.808	0.450-1.452	0.476
Lymphoma	2.703	1.186-6.162	0.108
Charlson Comorbidity index >3	1.742	1.023-2.965	0.041
Pitt bacteremia score ≥4	0.735	0.370-1.461	0.380
Relapsed or uncontrolled	1.048	0.825-1.332	0.700
Profound neutropenia	0.903	0.468-1.739	0.759
Previous chemotherapeutics	0.433	0.140-1.333	0.144
Hospital infection	1.052	0.512-2.160	0.890
Acute respiratory failure	1.153	0.796-1.670	0.451

### 3.3 Predictors of recurrent bloodstream infection at the onset of subsequent fever

We further analyzed patients with subsequent fever after initial BSI recovery, consisting of 92 non-RBSI cases and 135 RBSI cases(**Table 3**). Multivariable regression was used to adjust for baseline imbalances and screen independent predictors of recurrent infection. The analysis identified several independent predictors of RBSI during subsequent febrile episodes, including a CCI score >3 (OR = 3.031, 95% CI 1.059–8.674, *P* = 0.039), prior antibiotic exposure (OR = 6.603, 95% CI 3.064–14.230, *P* < 0.0001), acute respiratory failure (OR = 8.848, 95% CI 1.442–54.308, *P* = 0.019), and platelet count <10 × 10^9^/L (OR = 3.745, 95% CI 1.664–8.531, *P* = 0.002)(**Table 4**).

**Table 3:** Clinical features associated with RBSI and non-RBSI when suspected recurrent bloodstream infection.

Variables	non-RBSI group (n, %) n=92	RBSI group (n, %) n=135	P
Demographic information			
Age >55 years	22 (23.9)	20 (14.8)	0.035
Male sex	48 (52.2)	68 (52.3)	0.838
Underlying malignancies			
Acute myeloid leukemia	48 (52.2)	67 (49.6)	0.549
Acute lymphoblastic leukemia	21 (22.8)	48 (35.5)	0.035
Lymphoma	10 (10.9)	4 (3.1)	0.023
Other malignancies			
Disease status			
Relapsed or uncontrolled	45 (48.9)	74 (54.8)	0.103
Risk factors			
Charlson Comorbidity index >3	12 (13.0)	30 (22.2)	0.039
Pitt bacteremia score ≥4	8 (8.7)	30 (22.2)	0.023
Neutropenia	65 (70.7)	120 (88.9)	0.033
Profound neutropenia	56 (60.9)	98 (72.6)	0.046
Prolonged neutropenia	21 (22.8)	46 (34.1)	0.047
Previous chemotherapeutics	76 (82.6)	119 (88.1)	0.316
Hospital infection	79 (85.9)	78 (57.8)	0.039
Complications of dysfunctional organ systems			
Use of vasopressors	6 (6.5)	32 (23.7)	<0.001
Acute respiratory failure	2 (2.2)	23 (17.0)	<0.001
Renal insufficiency	2 (2.2)	10 (7.6)	0.030
Antibiotic therapy			
Prior antimicrobial exposure	31 (33.7)	100 (74.1)	<0.001
Use sensitive antibiotic	86 (93.5)	113 (83.7)	0.329
72h-IIAT	10 (10.9)	46 (34.1)	<0.001
Laboratory parameters			
Hemoglobin <70g/dL	46 (50.0)	98 (72.6)	<0.001
platelet count <10×10^9^/L	24 (26.1)	81 (52.9)	<0.001
Albumin <30g/L	23 (25.0)	72 (53.3)	<0.001
AST >120U/L	5 (4.2)	11 (8.1)	0.019
TBil >34.2µmol/L	7 (7.6)	20 (14.8)	0.014
PT >14s	6 (6.5)	25 (18.5)	<0.001

P < 0.05 indicates statistical significance.72h-IIAT, 72-hour inappropriate initial antimicrobial therapy; MDR-GNB, multidrug-resistant Gram-negative bacteria; ESBL-GNB, extended-spectrum β-lactamase-producing Gram-negative bacteria; CR-GNB, carbapenem-resistant Gram-negative bacteria; AST, aspartate aminotransferase; TBil, total bilirubin; PT, prothrombin time.

**Table 4:** Multivariable logistic regression for predicting confirmed RBSI among patients with suspected RBSI during subsequent febrile episodes.

Factor	Odds ratio	95% confidence interval	P value
Age >55 years	0.408	0.160-1.041	0.061
Charlson Comorbidity index >3	3.031	1.059-8.674	0.039
Hospital infection	0.156	0.062-0.394	<.001
Acute respiratory failure	8.848	1.442-54.308	0.019
Prior antimicrobial exposure	6.603	3.064-14.230	<.001
Platelet count <10×10^9^/L	3.745	1.644-8.531	0.002

### 3.4 Prognosis analysis in patients with RBSI

To identify risk factors for mortality after RBSI, patients were stratified by in hospital survival status into survivors (n = 136) and non-survivors (n = 37). Demographic characteristics, disease-related variables, organ function parameters, and antibiotic treatment regimens were included in univariable and multivariable regression analyses. As shown in **Table 5**, multivariable analysis identified several independent predictors of mortality, including 72-hour inappropriate initial antibiotic therapy (72-h IIAT) (OR = 7.464, 95% CI (2.485-22.415), *P*<0.001), total bilirubin ≥22 μmol/L (OR = 11.637, 95% CI (2.761-49.050), *P* = 0.001), use of vasopressors (OR = 12.405, 95% CI (3.988-38.593), *P* = 0.030), and respiratory failure (OR = 1.030-3.412, 95% CI (1.030-3.412), *P* = 0.040).

**Table 5:** Multivariable logistic regression of risk factors for mortality in patients with RBSI.

Factor	Odds ratio	95% confidence interval	P value
72h-IIAT	7.464	2.485-22.415	<0.001
Total bilirubin (TBil) ≥ 22 umol/l	11.637	2.761-49.050	0.001
Acute respiratory failure	1.874	1.030-3.412	0.040
Use of vasopressors	12.405	3.988-38.593	0.030

P < 0.05 indicates statistical significance.72h-IIAT, 72-hour inappropriate initial antimicrobial therapy.

### 3.5 Microbiological characteristics and resistance patterns of recurrent bloodstream infection

**Figure 3:**
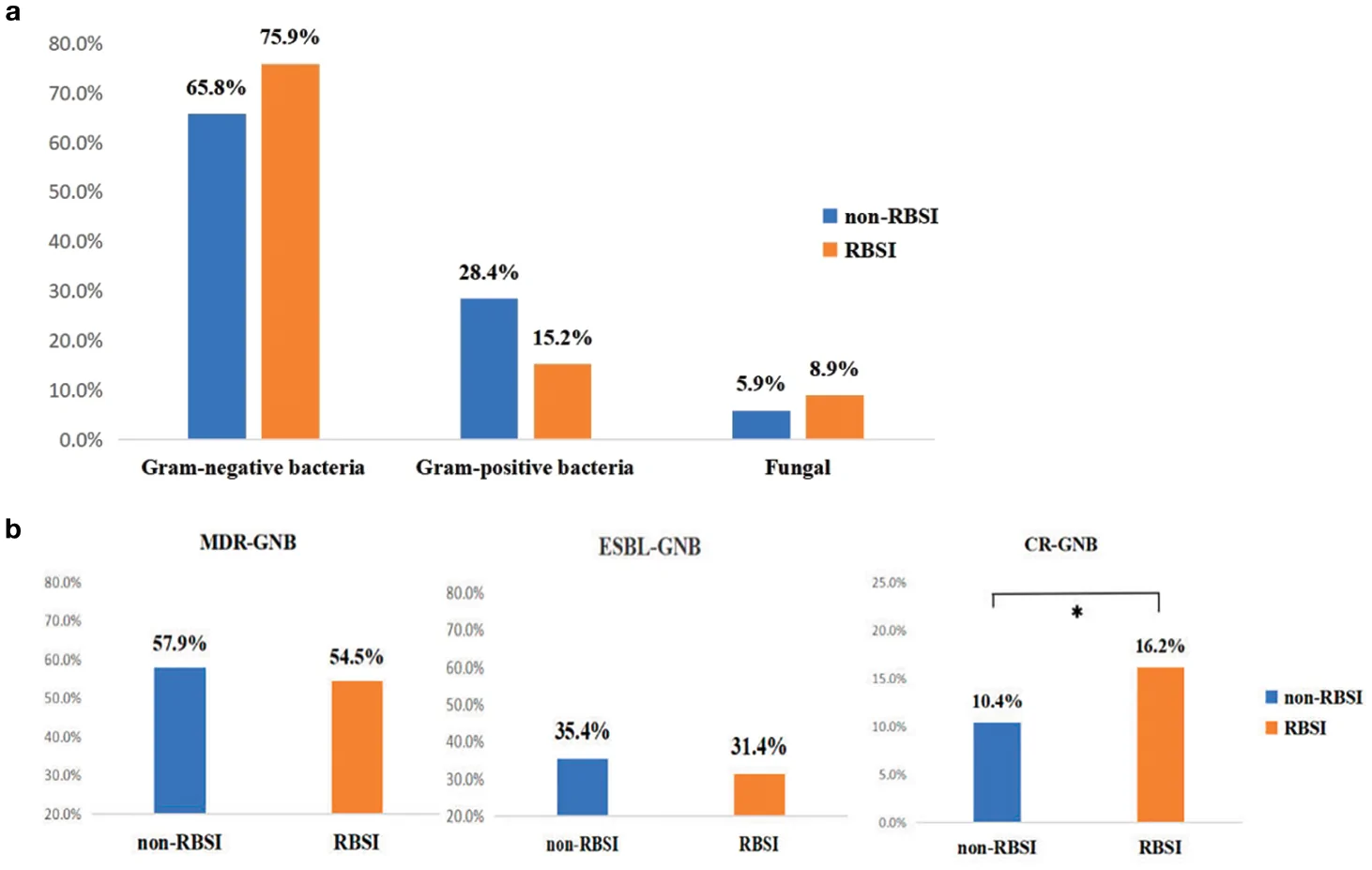
**(a)** Etiology of RBSI and non-RBSI. **(b)** Antibiotic resistance type of RBSI and non-RBSI (*p<0.05).

Given that early use of appropriate antibiotic therapy is associated with improved prognosis of HM patients with RBSI, to optimize clinical decision-making for patients with recurrent BSI, we analyzed the etiological characteristics of recurrent BSI. Microbiological profiles differed significantly between recurrent and non-recurrent BSI episodes. Gram-negative bacteria were more common in RBSI than in non–RBSI episodes (75.9% vs. 65.8%, P < 0.05), as were fungal pathogens (8.9% vs. 5.9%;**Figure 3A**; **Supplementary Table 1**). At the species level, Klebsiella pneumoniae was more frequently isolated in recurrent episodes (22.8% vs. 16.4%, P = 0.027), whereas coagulase-negative staphylococci were less common (27.5% vs. 57.5%, P < 0.001).

Among patients with RBSI, 54 of 173 (31.2%) had same-pathogen recurrence, while most recurrences were caused by different pathogens. The median interval to recurrence did not differ between same-pathogen and different-pathogen recurrence (86.5 vs. 81.0 days, P = 0.783).CR-GNB were significantly more common in recurrent episodes (16.2% vs. 10.4%, P = 0.032; **Figure 3B**).

## 4 Discussion

In this multicenter real-world cohort, recurrent bloodstream infection (RBSI) occurred in 15.0% of patients with hematological malignancies (HMs) and was associated with progressively increasing mortality. The 30-day in hospital mortality rose from 10.7% after the initial BSI episode to 19.0% after the first recurrence and 30.5% in patients with two or more recurrences. Importantly, we further addressed the clinically relevant question of early risk identification for RBSI. To this end, we assessed predictors of recurrence at two clinically relevant stages: during the initial BSI episode and during subsequent febrile episodes later in the treatment course, thereby providing a stage-specific framework for early recognition of RBSI in HMs.

Predictors of recurrence differed according to the timing of clinical assessment, highlighting the value of a dynamic rather than purely baseline-based approach (Maschmeyer et al., 2015; Woudt et al., 2018; Szubert et al., 2019; Froding et al., 2020). At the time of the initial BSI episode, the main clinical question is which patients are at higher risk of subsequent recurrence. In our study, a CCI >3 was the only independent predictor of RBSI, suggesting that comorbidity burden is an early marker of susceptibility to recurrence (Choi et al., 2021; Østergaard et al., 2023). This finding indicates that, even at the first episode of BSI, patients with high CCI scores deserve closer follow-up and greater clinical vigilance. We therefore further evaluated predictors of RBSI at the onset of subsequent fever after remission of the initial BSI. At this stage, CCI >3, prior antibiotic exposure, acute respiratory failure, and profound thrombocytopenia were independently associated with RBSI. Of note, we also identified an unexpected association in this cohort. An inverse correlation between hospital-acquired infection and recurrent infection was observed. This statistical association does not indicate protection or causality. Potential explanations include surveillance bias related to differences in monitoring intensity and timeliness of antimicrobial treatment between inpatient and outpatient settings, alongside residual confounding from unmeasured host and treatment factors, so it should be interpreted cautiously and requires further external validation. Consistently, patients with recurrence tended to have persistent or worsening organ dysfunction, whereas those without recurrence generally improved over time. These findings support a stage-specific approach to early recognition of RBSI in HMs.

Once RBSI has occurred, identifying patients at risk of poor outcome becomes critical. In our cohort, 72-hour inappropriate initial antibiotic therapy, hyperbilirubinemia, vasopressor use, and respiratory failure were independently associated with mortality after RBSI. Notably, this pattern was broadly consistent with our previous findings in initial BSI (Tang et al., 2018), highlighting the importance of early recognition and timely intervention in high-risk patients. It must be acknowledged that risk stratification of hematological malignancy (HM) patients with febrile neutropenia facilitates precise identification and intervention of high-risk patients with unfavorable prognosis (Kanter et al., 2025).

Our microbiological findings demonstrated that nearly 70% of recurrent episodes were caused by a different pathogen rather than the original one, consistent with the results of existing molecular research (Raven et al., 2018; Kim et al., 2023), indicating that empirical therapy for suspected RBSI should not simply repeat the regimen used for the initial episode, but should instead be guided by local epidemiology. In addition, CR-GNB were more common in recurrent episodes, underscoring the need to consider resistant pathogens. Because inappropriate early antibiotic therapy was associated with mortality (Tang et al., 2020), prompt microbiological investigation remains essential. Empirical treatment should be initiated according to local pathogen distribution and resistance patterns, and then refined once microbiological results become available. Approximately one-third of recurrent BSI episodes were caused by pathogens of the same species identified at initial infection. Patients with hematological malignancies often have sustained immunosuppression, and opportunistic pathogens may persistently colonize even after effective antimicrobial therapy. Therefore, recovery of identical species at recurrence cannot simply be interpreted as persistent infection, catheter-related infection, inadequate source control or true relapse. Prior molecular typing studies demonstrate that only a minority of such isolates are genetically identical to the initial strain. Without stored isolates for genomic analysis in this retrospective work, we compared susceptibility profiles of paired isolates; concordant results were found in only half of pairs, indirectly suggesting that many recurrent infections stem from distinct strains. We recognize that susceptibility data serve merely as surrogate evidence. Future prospective studies with whole-genome sequencing are required to distinguish true relapse from *de novo* reinfection in this vulnerable population.

This study presents the largest cohort with long-term follow-up investigating recurrent BSIs in HM patients, providing comprehensive clinical data for suspected recurrent BSIs. Several limitations should be noted. First, patients who died within 7 days after initial BSI were excluded from the primary analysis, as they lacked sufficient observation time to develop recurrent infection. This approach removes the cohort of most critically ill patients, which may underestimate the overall incidence of R-BSI and potentially attenuate the magnitude of identified risk factors. Nevertheless, sensitivity analysis incorporating these early-death subjects into the non-R-BSI group yielded consistent findings. Second, The subgroup analysis comparing initial BSI and subsequent febrile episodes excluded patients with two or more recurrent BSI to enhance group comparability, potentially introducing selection bias. Given that patients with multiple recurrences carry sustained immunosuppression and repeated antimicrobial exposure, these results are not generalizable to this high-risk population. Third, follow-up duration varied widely across participants. Patients with limited follow-up time had fewer opportunities to develop recurrent BSI, which could lead to unequal event ascertainment. However, 87.9% of recurrent bloodstream infections occurred within 1 year of the initial episode, and sensitivity analysis restricted to patients with recurrence within 365 days confirmed consistent findings with the primary analysis.

In summary, recurrent BSI is common in HM patients and its association with increased mortality in HMs. Our study systematically explored the clinical characteristics and time-specific predictive factors of recurrent BSI in HM patients. CCI >3 at initial BSI and adverse clinical features during subsequent fever may facilitate early identification of patients at high risk of recurrence and support more timely, targeted management. Prospective studies are needed to further validate these findings.

## Data Availability

The data analyzed in this study is subject to the following licenses/restrictions: Data will be made available from the corresponding author upon reasonable request and with prior written approval. Requests to access these datasets should be directed to lixiner1975@163.com.
