# Supplementary material for: Predictors and outcomes of recurrent bloodstream infection in hematological malignancies: a multicenter real-world study
Source: Front Cell Infect Microbiol. 2026 Sep 1;16:1874798. doi: 10.3389/fcimb.2026.1874798 (PMC13574583; doi:10.3389/fcimb.2026.1874798)
Supplement: Supplementary file 1 [file Table1.docx]

**Supplement Table 1.** Etiology of initial and recurrent BSIs

| Microorganism | Initial BSI  N (isolates) = 1174,  n (%) | Recurrent BSI  N (isolates) = 224,  n (%) | P |
| --- | --- | --- | --- |
| **Gram-negative bacteria** | 772(65.8) | 170(75.9) | ＜0.001 |
| Enterobacter | 573(54.7) | 114(59.7) | 0.235 |
| *Escherichia coli* | 318(27.1) | 58(25.9) | 0.743 |
| *Klebsiella pneumoniae* | 193(16.4) | 51(22.8) | 0.027 |
| *Enterobacter cloacae* | 36(3.1) | 5(2.2) | 0.498 |
| *Non-fermentative bacteria* | 205(19.6) | 47(24.6) | 0.118 |
| *Pseudomonas aeruginosa* | 97(8.3) | 14(6.3) | 0.307 |
| *Acinetobacter baumannii* | 11(0.9) | 9(4.0) | ＜0.001 |
| Stenotrophomonas maltophilia | 19(1.6) | 5(2.2) | 0.517 |
| **Gram-positive bacteria** | 333(28.4) | 34(15.2) | ＜0.001 |
| Coagulase-negative staphylococci | 161(57.5) | 14(27.5) | ＜0.001 |
| *Staphylococcus aureus* | 51(15.5) | 5(6.9) | 0.057 |
| Enterococcus | 28(8.9) | 6(8.2) | 0.849 |
| Streptococcus | 74(21.6) | 9(12.3) | 0.071 |
| **Fungal** | 69(5.9) | 20(8.9) |  |
| Candida | 67(97.1) | 20(100) | 0.892 |
| Other Fungal | 2(2.9) | 0 |  |
| **MDR** bacteria | 607(57.9) | 104(54.5) | 0.373 |
| **ESBL-**producing Enterobacterales | 202(35.4) | 38(31.4) | 0.157 |
| **CR-GNB** | 99(10.2) | 20(16.7) | 0.019 |
| **CR-KP** | 14(1.3) | 4(2.1) | 0.420 |

Note: MDR bacteria，multi-drug resistance bacteria; ESBL, extended-spectrum beta-lactamase; CR-GNB, Carbapenem-resistant gram-negative bacteria; CR-KP, Carbapenem-resistant klebsiella pneumoniae.

Supplementary Table 2 Patients with recurrent BSI: Comparison of characteristic between index BSI and recurrent BSI

| Variables | index BSI(n, %),n=135 | recurrent BSI (n, %),n=135 | P |
| --- | --- | --- | --- |
| **Demographic information** |  |  |  |
| Age >55 years | 18(13.3) | 20(14.8) | 0.765 |
| Male sex | 76(56.3) | 68(52.3) | 0.838 |
| **Underlying malignancies** |  |  |  |
| Acute myeloid leukemia | 76(56.3) | 67(49.6) | 0.549 |
| Acute lymphoblastic leukemia | 44(32.6) | 48(35.5) | 0.532 |
| Lymphoma | 6(4.4) | 4(3.1) | 0.618 |
| Other malignancies |  |  |  |
| **Disease status** |  |  |  |
| Relapsed or uncontrolled | 77(57.0) | 74(54.8) | 0.451 |
| **Risk factors** |  |  |  |
| Charlson Comorbidity index >3 | 11(8.1) | 14(10.4) | 0.548 |
| Pitt bacteremia score ≥4 | 24(17.8) | 30(22.2) | 0.133 |
| Neutropenia | 127(94.1) | 120(88.9) | 0.533 |
| Profound neutropenia | 105(77.8) | 98(72.6) | 0.851 |
| Prolonged neutropenia | 45(33.3) | 46(34.1) | 0.451 |
| Previous chemotherapeutics | 124(91.8) | 119(88.1) | 0.516 |
| Hospital infection | 118(87.4) | 78(57.8) | 0.039 |
| **Complications of dysfunctional organ systems** |  |  |  |
| Use of vasopressors | 20(14.8) | 32(23.7) | 0.034 |
| Acute respiratory failure | 21(15.6) | 23(17.0) | 0.146 |
| Renal insufficiency | 2(1.5) | 10(7.6) | 0.030 |
| **Antibiotic therapy** |  |  |  |
| Prior antimicrobial exposure | 98(72.6) | 100(74.1) | 0.579 |
| Use sensitive antibiotic | 122(90.4) | 113(83.7) | 0.329 |
| 72h-IIAT | 97(71.9) | 89(65.9) | 0.212 |
| **Laboratory parameters** |  |  |  |
| Hemoglobin <70g/dL | 104(77.0) | 98(72.6) | 0.226 |
| Platelet <10×10^3^mm^-3^ | 84(62.2) | 81(52.9) | 0.040 |
| Albumin <30g/L | 70(51.8) | 72(53.3) | 0.668 |
| AST >120U/L | 13(9.6) | 11(8.1) | 0.409 |
| TBil >34.2µmol/L | 24(17.8) | 20(14.8) | 0.631 |
| PT >14s | 29(21.5) | 25(18.5) | 0.354 |

Supplementary Table 3 Patients with non-RBSI：Comparison of characteristic between Initial BSI and Subsequently febrile episodes but without BSI

| Variables | index BSI (n, %)  n=92/264 | Subsequently febrile episodes but without BSI (n, %)n=92 | P |
| --- | --- | --- | --- |
| **Demographic information** |  |  |  |
| Age >55 years | 83(22.0) | 22(23.9) | 0.675 |
| Male sex | 151(54.4) | 48(52.2) | 0.838 |
| **Underlying malignancies** |  |  |  |
| Acute myeloid leukemia | 140(53.0) | 48(52.2) | 0.549 |
| Acute lymphoblastic leukemia | 51(19.3) | 21(22.8) | 0.332 |
| Lymphoma | 30(11.4) | 10(10.9) | 0.618 |
| Other malignancies |  |  |  |
| **Disease status** |  |  |  |
| Relapsed or uncontrolled | 164(62.1) | 45(48.9) | 0.024 |
| **Risk factors** |  |  |  |
| Charlson Comorbidity index >3 | 39(14.8) | 12(13.0) | 0.545 |
| Pitt bacteremia score ≥4 | 58(21.9) | 8(8.7) | ＜0.001 |
| Neutropenia | 230(87.1) | 65(70.7) | 0.028 |
| Profound neutropenia | 139(52.7) | 56(60.9) | 0.046 |
| Prolonged neutropenia | 65(24.6) | 21(22.8) | 0.451 |
| Previous chemotherapeutics | 217(82.2) | 76(82.6) | 0.716 |
| Hospital infection | 148(56.1) | 79(85.9) | ＜0.001 |
| **Complications of dysfunctional organ systems** |  |  |  |
| Use of vasopressors | 59(22.3) | 6(6.5) | ＜0.001 |
| Acute respiratory failure | 35(13.3) | 2(2.2) | ＜0.001 |
| Renal insufficiency | 13(4.9) | 2(2.2) | 0.130 |
| **Antibiotic therapy** |  |  |  |
| Prior antimicrobial exposure | 166(62.9) | 58(63.0) | 0.479 |
| Use sensitive antibiotic | 222(84.1) | 86(93.5) | 0.069 |
| 72h-IIAT | 188(71.2) | 82(89.1) | 0.012 |
| **Laboratory parameters** |  |  |  |
| Hemoglobin <70g/dL | 205(77.7) | 46(50.0) | ＜0.001 |
| Platelet <10×10⁹/L | 113(42.8) | 24(26.1) | ＜0.001 |
| Albumin <30g/L | 115(43.6) | 23(25.0) | ＜0.001 |
| AST >120U/L | 22(8.3) | 5(4.2) | 0.059 |
| TBil >34.2µmol/L | 43(16.3) | 7(7.6) | 0.022 |
| PT >14s | 58(22.0) | 6(6.5) | ＜0.001 |

**Supplementary Table 4** Clinical features about patient died with in 7 days of primary BSI

| Variables | Patient died with in 7 days of primary BSI (n, %) n=133 |
| --- | --- |
| **Demographic information** |  |
| Age(mean ± SD) | 46.36±15.52 |
| Age >55 years | 46(34.6) |
| Male sex | 80(60.2) |
| **Underlying malignancies** |  |
| Acute myeloid leukemia | 71(53.4) |
| Acute lymphoblastic leukemia | 30(22.6) |
| Lymphoma | 17(12.8) |
| Other malignancies | 19(13.9) |
| HSCT | 4(3.0) |
| **Disease status** |  |
| Relapsed or uncontrolled | 117(88.0) |
| **Risk factors** |  |
| Charlson Comorbidity index >3 | 32(24.1) |
| Pitt bacteremia score ≥4 | 94(70.7) |
| MASCC＜21 | 126(94.7) |
| Neutropenia | 122(91.7) |
| Profound neutropenia | 107(80.5) |
| Prolonged neutropenia | 19(14.3) |
| Previous chemotherapeutics | 102(76.7) |
| Receipt of corticosteroids | 66(49.6) |
| Hospital infection | 90(67.6) |
| central venous catheters | 42(31.6) |
| urinary catheters | 16(12.0) |
| **Complications of dysfunctional organ systems** |  |
| Use of vasopressors | 84(63.2) |
| Acute respiratory failure | 84(63.2) |
| Renal insufficiency | 10(7.5) |
| **Antibiotic therapy** |  |
| Prior antimicrobial exposure | 91(68.4) |
| Use sensitive antibiotic | 94(68.6) |
| 72h-IIAT | 74(55.6) |
| **Bacteria resistance** |  |
| MDR-GNB | 82(59.9) |
| ESBL-GNB | 46(33.5) |
| CR-GNB | 20(15.0) |
| **Laboratory parameters** |  |
| Hemoglobin <70g/dL | 122(91.7) |
| Platelet <10×10⁹/L | 86(64.7) |
| Albumin <30g/L | 97(72.9) |
| AST >120U/L | 31(23.3) |
| TBil >34.2µmol/L | 38(28.6) |
| PT >14s | 63(47.4) |

**Supplementary Table 5** Sensitivity analysis for risk factors of RBSI at the initial BSI

| **Factor** | **Hazard Ratio** | **95% Confidence Interval** | **P Value** |
| --- | --- | --- | --- |
| Age >55 years | 0.752 | 0.414–1.366 | 0.349 |
| Lymphoma | 2.928 | 1.340–6.396 | 0.011 |
| Charlson Comorbidity index >3 | 1.667 | 0.985–2.820 | 0.047 |
| Pitt bacteremia score ≥4 | 0.649 | 0.327–1.288 | 0.217 |
| Profound neutropenia | 0.773 | 0.434–1.376 | 0.382 |
| Relapsed or uncontrolled disease | 1.212 | 0.766–1.918 | 0.411 |
| Hospital infection | 1.169 | 0.559–2.444 | 0.678 |
| Previous chemotherapeutics | 0.546 | 0.212–1.406 | 0.210 |
| Acute respiratory failure | 1.169 | 0.559–2.444 | 0.183 |
